# A bundle of the top 10 OPAT publications in 2024

**DOI:** 10.1017/ash.2025.10281

**Published:** 2026-01-13

**Authors:** Lindsey M. Childs-Kean, Sara F. Azimi, Alison M. Beieler, Laila Castellino, Sara C. Keller, Margaret Pertzborn, Alexandra Yamshchikov, Leah H. Yoke, Kathleen Young, Monica V. Mahoney

**Affiliations:** 1 Department of Pharmacy Education and Practice, College of Pharmacy, University of Florida, PO Box 100486, Gainesville, FL, USA; 2 Department of Pharmacy, Nebraska Medicine, Omaha, NE, USA; 3 Harborview Medical Center, Seattle, WA, USA; 4 Division of Infectious Disease and Geographic Medicine, UT Southwestern Medical Center, Dallas, TX, USA; 5 Parkland Health, Dallas, TX, USA; 6 Division of Infectious Diseases, Department of Medicine, Johns Hopkins University School of Medicine, Baltimore, MD, USA; 7 Department of Health Policy and Management, Johns Hopkins Bloomberg School of Public Health, Baltimore, MD, USA; 8 Department of Pharmacy, Mayo Clinic Health System-Northwest Wisconsin Region, Eau Claire, WI, USA; 9 Division of Infectious Diseases, University of Rochester Medical Center, Rochester, NY, USA; 10 Vaccine and Infectious Disease Division, Fred Hutch Cancer Center, Division of Allergy and Infectious Diseases, Department of Medicine, University of Washington School of Medicine, Seattle, WA, USA; 11 Division of Infectious Diseases, Oregon Health & Science University, Portland, OR, USA; 12 Division of General Internal Medicine and Geriatrics, Section of Addiction Medicine, Oregon Health & Science University, Portland, OR, USA; 13 Department of Pharmacy, Beth Israel Deaconess Medical Center, Boston, MA, USA

## Abstract

**Objective:**

Outpatient parenteral antimicrobial therapy (OPAT) is a mainstay of clinical infectious diseases practice, and OPAT-related publications continue to be prominent in journals. The objective of this article is to summarize ten clinically important OPAT-related publications from 2024.

**Design:**

Narrative review.

**Methods:**

Eighty-one articles were found in a literature search, and 56 met inclusion criteria. A survey containing 25 articles was sent to an email listserv of clinicians with OPAT experience.

**Results:**

This article summarizes the top 10 OPAT articles published in 2024, based on those survey results.

**Conclusions:**

Common themes from the top 10 OPAT articles published in 2024 included OPAT clinician workload, patient perspectives of OPAT, tools for OPAT work, and dalbavancin use.

## Introduction

Outpatient parenteral antimicrobial therapy (OPAT) and complex outpatient antimicrobial therapy (COpAT) are methods of delivering ongoing care and support to patients receiving antimicrobials outside the acute care setting.^1,2^ To keep abreast of OPAT and COpAT publications, we summarize 10 important OPAT publications from 2024 as voted on by a national multidisciplinary group of OPAT clinicians. Prior yearly Top 10 lists have been published since 2022.^3–5^

## Methods

**Figure 1.**
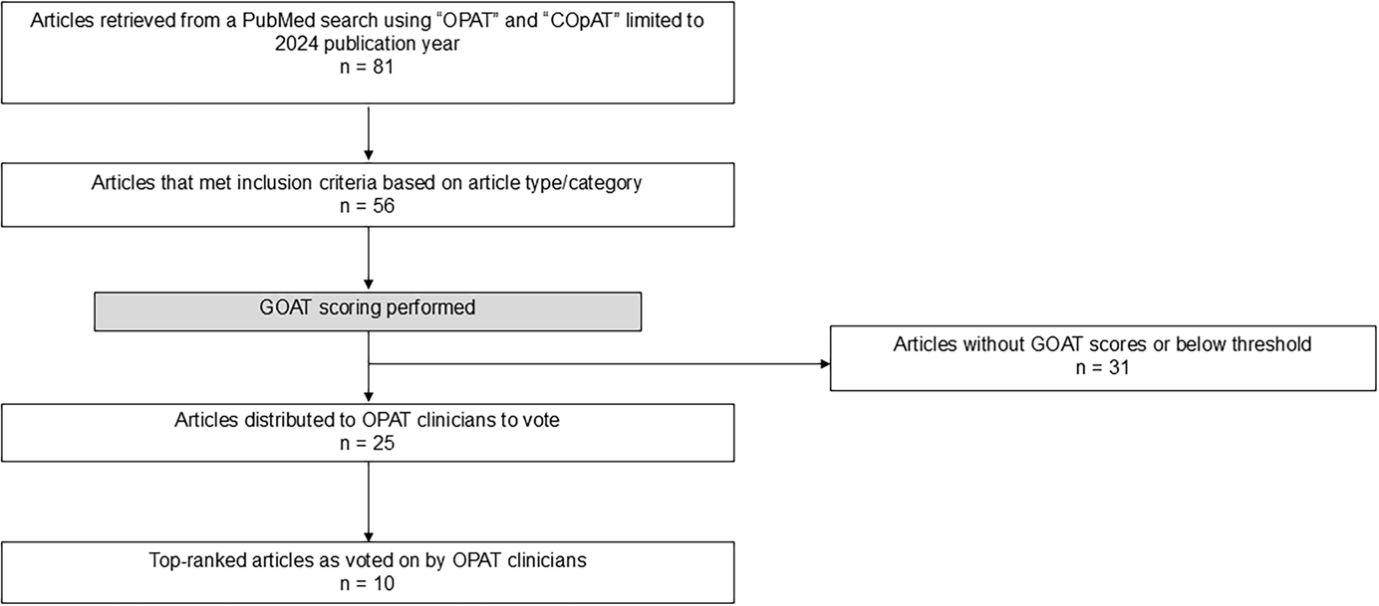
Selection of articles. Abbreviations: COpAT, complex outpatient antimicrobial therapy; GOAT, grading outcomes–based research in antimicrobial therapy; OPAT, outpatient parenteral antimicrobial therapy.

PubMed-indexed publications with Medline key terms “OPAT” and “COpAT” published between 1 January and 31 December 2024 were identified. Articles were reviewed to ensure publication in 2024 and in English. Narrative reviews without new data, opinion pieces, and in vitro-only studies were excluded. 81 publications were identified, with 56 meeting inclusion criteria and were assigned a Grading Outcomes-based research in Antimicrobial Therapy (GOAT) score.^3^ Briefly, the GOAT score incorporates the journal’s impact factor, the article’s number of citations, and time between publication and date the GOAT score was calculated. The twenty-five articles with the highest GOAT score were included in a survey to determine the top 10 list. See Figure 1 for details of the article selection process.

The survey containing the twenty-five studies listed the articles in alphabetical order by first author’s last name and included a link to each study; this was sent to a national email listserv of 294 clinicians who expressed interest and practice experience in OPAT. Except for the two authors who calculated the GOAT scores and built the survey, the survey recipients were blinded to the GOAT scores. Survey recipients were asked to select the ten publications that were most impactful to their OPAT/COpAT practice in terms of clinical practice applicability, feasibility, and innovation. 73 survey responses were received (25% response rate). The top ten articles are presented both in Table 1 and below alphabetically by the first author’s last name and divided into programmatic studies, those that investigated the impact of OPAT programs on different outcomes, and therapy-based studies, which compared outcomes between therapies.

**Table 1.** Summary of top OPAT publications from 2024

Citations	Study design	Primary and secondary outcomes with results	Strengths/limitations/potential impacts
Programmatic studies			
Bouzigard et al^7^	Pre-post study looking at outcomes for OPAT patients after a reduction in clinical staff	Primary Outcome: ED visits within 30 days of hospital discharge increased from 20.4% during the higher-staffed period to 34% during the lower-staffed period Secondary Outcomes: Readmissions within 30 days of hospital discharge increased from 8.6% during the higher-staffed period to 17.9% during the lower-staffed period VAD-related complications did not increase during lower-staffed period (total of 39 complications)	Strengths: Unique study design, looking at impacts after an intervention is removed Limitations: Single center study; may limit generalizability Potential Impacts: Emphasizes importance of maintaining staffing for OPAT programs, particularly in a safety-net hospital, to prevent hospitalizations, ED visits, and VAD complications
Canterino et al^8^	Retrospective cohort of the development of an OPAT registry	Primary Outcome: Automated OPAT registry in the EMR was successfully implemented across the health system, capturing 3,956 unique patients and 4,710 OPAT episodes over four years Secondary Outcomes: Patient identification accuracy by registry: 93.8% All variables included in registry demonstrated>90% accuracy	Strengths: Large sample size, demonstrated high registry accuracy, report detailed to increase reproducibility. Limitations: Registry dependent on Epic EMR build, reliance on provider documentation (SmartList for complications, Episode of Care for enrollment) Potential impacts: Reproducible model for other health systems to build from, enables program to track OPAT metrics
Hassanin et al^9^	Retrospective study to identify predictors of successful OPAT in patients with osteomyelitis from a diabetic forefoot infection	Primary Outcome: Treatment success of osteomyelitis in diabetic forefoot infection with OPAT was 63.6% at 12 months Secondary outcomes: Predictors of treatment failure: peripheral arterial disease (*P* = .005) and poor glycemic control (HbA1c > 6.5%, *P* = .025)	Strengths: Moderate cohort size, analyzed OPAT treatment outcomes combined with multidisciplinary approach, identified predictors of treatment failure Limitations: Single-center study, may limit generalizability; retrospective review of data limits assessment of some patient-specific factors that could influence healing outcomes; exclusion of surgery- related factors that could influence healing outcomes; exclusion of the effects of revascularization procedures in the setting of repeated OPAT courses Potential impacts: Highlights OPAT treatment success in patients with osteomyelitis from a diabetic forefoot infection
Mohammed et al^10^	Systematic review and meta-analysis of barriers and facilitators to OPAT services	Primary Outcomes: OPAT was more cost-effective and had higher levels of patient satisfaction than inpatient care, with similar rates of efficacy Barriers included proper patient selection, poor communication, lack of support, and out of pocket costs	Strengths: Thematic analysis of papers by organizing into domains of topic; identification of barriers and facilitators influencing implementation and expansion of OPAT in a broad number of publications; bias evaluation performed Limitations: Subject to bias given selection process; inclusion of studies of a variety of designs with multiple reviewers could result in inconclusive results; studies primarily conducted in Europe and North America Potential impacts: Gives a broad overview of publications highlighting barriers and facilitators for OPAT care
Mohammed et al ^11^	Systematic review and meta-analysis comparing inpatient treatment to OPAT	Primary Outcome: Mortality RR .54, 95% CI .23–1.26, *P* = .16, I^2^ 18%. Secondary Outcomes: Treatment failure (RR 1.0, 95% CI .59–1.72, *P* = .99, I^2^ 38%) ADRs (RR .89, 95% CI .69–1.15, *P* = .38, I^2^ 0%) Device complications (RR .58, 95% CI .17–1.98, *P* = .39, I^2^ 0%)	Strengths: Used PRISMA guidelines, evaluated bias, used GRADE criteria Limitations: Few trials included, limited disease states, varied geographic locations, different antibiotic regimens used inpatient versus outpatient, endpoints reported differently across trials Potential Impacts: Reinforces safety and efficacy of OPAT
Munsiff et al^12^	Description of EHR modification (Epic) to collate multiple pieces of data including labs, microbiology, imaging, infusion details, ID notes, TDM, IV access device oversight and timely follow-up visits into a single screen, to facilitate oversight of a large OPAT patient population	Primary Outcomes: Captured 3,402 OPAT episodes over four years, allowed for efficient (< 3 h/wk) multidisciplinary rounds to oversee 130–145 patients per week Reduced cognitive load and rounding time while facilitating consistent TDM, IV access device oversight, and timely follow-up	Strengths: Demonstrated the usefulness of modifying the EHR to have all the required information in one place, allowing for efficient rounding, standardized order sets, and proactive lab tracking; reduced missed TDM and VAD-related complications. Limitations: The modifications described are specific to Epic and may not be translatable to other EHRs. Potential Impacts: Modifications described can be adopted by other institutions that use Epic. The extractable SmartForm data enabled workload analytics and program justification.
Peter et al^13^	Explanatory- sequential mixed- methods (Survey then in-depth interviews of patients receiving OPAT during a pilot phase in one region in Germany)	Primary Outcome: All respondents had a positive opinion of OPAT and almost all would choose OPAT again Secondary Outcomes: Infrequently reported problems included material storage and feeling uncomfortable with catheter Patients reported better quality of life doing treatment at home but anxiety at the beginning of treatment and cost concerns	Strengths: Mixed methods study, interviews gave deeper insight into true patient experience Limitations: One area in Germany; experiences may not be fully generalizable Potential Impacts: OPAT has been shown to be widely acceptable by patients. Additional patient education could be helpful before discharge with OPAT
Schranz et al^22^	Prospective observational time-in-motion study with retrospective descriptive analysis of unbilled clinical time spent caring for OPAT patients	Primary Outcomes: Median OPAT time: 27 min/wk per episode outside visits Vancomycin regimens: 40 min/wk median OPAT time outside visits Non-Vancomycin regimens: 23 min/wk median OPAT time outside visits 73% of episodes qualified for Level III billing per OPAT week Secondary Outcomes: Median OPAT duration 35 days 12%, 48%, and 40% with 0, 1, ≥ 2 follow up visits per OPAT course	Strengths: Primary focus of capturing nonbillable workload associated with OPAT care; ability to differentiate by regimen composition Limitations: Focus on electronic documentation underestimates other modalities of OPAT labor - telephone, fax, in person; self report time-in-motion method may limit external validity, recall bias; single site study nested in specific program structure and patient panel composition may limit generalizability Potential Impacts: The burden on non-billable labor of OPAT is significant, issues a call to action for redefining compensation structures for OPAT services
Therapy-based studies			
Hamad et al^26^	Retrospective cohort comparing outcomes between ceftriaxone and SOC (cefazolin, nafcillin, or oxacillin) for MSSA BSI	Primary outcome: Infection-related 90-day readmission similar: 17% ceftriaxone vs 20% SOC (aOR .89 [95% CI .67–1.18]) Secondary outcome: All-cause 90-day readmission similar: 25% ceftriaxone vs 29% SOC (aOR .79 [95% CI .62–1.03])	Strengths: Large cohort size; compared ceftriaxone to guideline-recommended treatment for MSSA BSI; included a large variety of index infections Limitations: Exclusion of patients older than 64 years; did not report dosing of ceftriaxone regimens; did not compare treatment-related safety outcomes between groups Potential impacts: Ceftriaxone is a treatment option in low-risk or uncomplicated cases of MSSA BSI
Streifel et al^28^	Retrospective cohort describing the use of dalbavancin for the treatment of vertebral osteomyelitis	Primary Outcome: Successful course completion utilizing dalbavancin (to complete 6 wk therapy) in 29 of 34 patients in a cohort with 79% substance use Secondary outcomes: 30-day relapse/recurrence in 2 patients 90-day relapse/recurrence in 1 patient	Strengths: Large cohort of vertebral osteomyelitis patients, including those with substance use, not all of which had source control; large number of MRSA isolates included Limitations: Limited patient follow-up Potential Impacts: Dalbavancin is an encouraging alternative for vertebral osteomyelitis

Abbreviations: OPAT, outpatient parenteral antimicrobial therapy; ED, emergency department; VAD, vascular access device; EMR, electronic medical record; HbA1C, hemoglobin A1C; RR, risk ratio; CI, confidence interval; ADRs, adverse drug reactions; EHR, electronic health record; ID, infectious diseases; TDM, therapeutic drug monitoring; IV, intravenous; COpAT, complex outpatient antimicrobial therapy; SOC, standard of care; MSSA, methicillin-susceptible *Staphylococcus aureus*; BSI, bloodstream infection; aOR, adjusted odds ratio; MRSA, methicillin-resistant *Staphylococcus aureus*.

## Publication summaries

### Programmatic studies

#### Outpatient parenteral antimicrobial therapy in a safety net hospital: opportunities for improvement

In 2009, Parkland Health in Dallas, TX, USA developed an innovative, award-winning self-administration OPAT (S-OPAT) model for uninsured patients.^6^ The OPAT program has evolved to care for insured patients who receive OPAT via home infusion as well as patients receiving OPAT at hemodialysis centers or in skilled nursing facilities. Unfortunately, after COVID, significant attrition occurred across the OPAT team, including four of six nurses, a care coordinator, and a nurse navigator, while the infectious diseases (ID) pharmacist, physicians, and advanced practice providers were tasked with additional responsibilities. This provided a unique opportunity to determine the impact of reducing OPAT program staff on 30-day emergency department (ED) visits, 30-day hospitalizations, and vascular access device (VAD) complications. In this study,^7^ 186 patients received OPAT in the predisruption period (April–June 2021) and 156 received OPAT in the postdisruption period (January–March 2022). ED visits increased in the postdisruption period from 20 to 34% (*P* = .001). Readmissions also increased, from 9% to 18% (*P* = .01). The hazard ratio for VAD complications did not increase (*P* = .07). This article describes what happens when a program designed to care for vulnerable patients requiring OPAT undergoes staffing disruptions: healthcare utilization increases. Fully staffing OPAT programs is important in preventing unnecessary healthcare utilization.

#### Creation and validation of an automated registry for outpatient parenteral antibiotics

This study^8^ at one center in the USA developed an automated OPAT registry within the electronic health record (EHR), specifically Epic^TM^, using “Episode of Care” enrollment, initiated by the ID consultant at the time of finalizing the antibiotic plan. The primary outcome was to create a sustainable, EHR-based registry and secondary outcome to validate it against formal record review.

The registry was successfully implemented across the health system, capturing 3,956 unique patients and 4,710 OPAT episodes over four years. In the validation convenience sample of 146 antibiotic episodes, 137 were included demonstrating a registry accuracy of 93.8% for identifying eligible patients. All variables, age, sex, race/ethnicity, primary payor, discharge disposition, antibiotic, infection syndrome, 30-day readmission, 30-day mortality, and complications via OPAT SmartList, demonstrated>90% accuracy. The OPAT program saved an estimated 88,820 hospital days, or roughly 22,000 days annually.

This study demonstrates that an EHR-based OPAT registry can provide highly accurate, automated capture of patient demographics, outcomes, and programmatic value. Because Epic^TM^ and similar EHRs are widely used, this methodology is reproducible at other health systems. Such registries not only streamline data collection, a known barrier, but also provide metrics to support evidence of OPAT’s value.

#### Predictors of successful antibiotic treatment of osteomyelitis in diabetic forefoot infection

Diabetic foot infections (DFI) are prevalent, yet treatment management is varied. This retrospective study^9^ in Ireland analyzed patients admitted with acute DFI from 2016 to 2020 who were assessed by a multidisciplinary team and then treated with a prolonged OPAT course. Exclusion criteria included infections of non-diabetic feet, heel/tarsal bones, or needing urgent surgical intervention and those with chronic ulcerations. Outcomes were successful treatment of osteomyelitis with healing, no recurrence, and no amputations at 12 months.

Of 264 infected toes, 168 (63.6%) achieved full healing at 12 months. Of the 96 toes that failed, 92 (34.8%) required amputation, 86 (32.6%) of which being minor amputations. 43 toes that weren’t healed received a second OPAT course without healing. Through multivariate analysis, peripheral arterial disease (PAD) and poor glycemic control were most statistically significant for poor treatment outcomes; OR 2.78 (95% CI = 1.36–5.69; *P* = .005) and OR 2.46 (95% CI = 1.12–5.38; *P* = .025) respectively.

The authors highlight OPAT and a multi-disciplinary treatment strategy as a promising approach to managing osteomyelitis in diabetic foot ulcers. This study confirmed the risk factors associated with treatment failure include PAD and poor glycemic control and indicates additional antibiotic courses are not beneficial. Future research could evaluate other patient care related outcomes.

#### Barriers and facilitators for the implementation and expansion of outpatient parenteral antimicrobial therapy: a systematic review

As OPAT programs have expanded in both capacity and reach, a systematic review was conducted to understand, within a broader context, barriers and facilitators to successful OPAT programs.^10^ The authors performed a systematic manual literature review with inclusion criteria of studies which described intravenous (IV) antimicrobial administration in an outpatient setting, facilitating factors for OPAT, and barriers to OPAT services. A total of 8,754 studies were preliminarily extracted from databases, with 514 studies reviewed in detail. Ultimately, 147 studies were included in the study. Most studies were performed in North America (44.2%) followed by Europe (32%). Only 14% of the studies included a comparator group; most were retrospective (68.4%).

Using the Consolidated Framework for Implementation Research (CFIR) theoretical framework, data from each study was organized into five domains including innovation; the inner setting referring to the institution providing OPAT whether home, infusion center, or hospital; the outer setting describing external factors that might affect OPAT implementation; the individual or those whose role included OPAT engagement, and the implementation process. Each study was evaluated for each domain and was manually coded and categorized.

Overall findings suggest that OPAT was safe and effective, with no studies suggesting inferiority of OPAT-based approaches when compared to conventional. Further, OPAT brought significant cost savings, with estimates ranging from 30 to 75%, noted primarily by shortening length of hospital stay. However, for patients without private health insurance, out of pocket costs could be high. Coordination and communication within an OPAT program were noted by multiple studies as a potential pitfall, as the workload for both providers and support staff was identified as a potentially limiting factor in OPAT expansion. Nearly all studies evaluated showed substantial preference for OPAT over conventional inpatient treatment, with most patients within the analyses recommending it to others. However, if patients had no caregiver, poor mobility, or a lower income level, they were likely to prefer inpatient management. Patients receiving OPAT with home healthcare had higher satisfaction than those at a skilled nursing facility. This study provides a broad and sweeping overview of OPAT implementation, providing a pathway for future OPAT expansion and longevity.

#### Safety and efficacy of outpatient parenteral antimicrobial therapy: a systematic review and meta-analysis of randomized clinical trials

This study^11^ was a systematic review and meta-analysis of randomized controlled trials (RCTs) comparing adult and pediatric patients receiving IV antimicrobials in the outpatient setting to the inpatient setting. Risk ratios were calculated for mortality, treatment failure, adverse drug reactions (ADRs), and device complications.

Thirteen studies involving 1,310 patients (1,363 OPAT episodes) were included. Febrile neutropenia was the most common disease state included (*N* = 5, 2 in adults, 3 in children), followed by cellulitis (*N* = 2, 1 each in adults and children). Others included severe intravenous drug use (IVDU)-related infections, peritonsillar abscess, *Staphylococcus aureus* bacteremia/endocarditis, cystic fibrosis, and others. Notably, antibiotic regimens differed in inpatient versus outpatient settings. Additionally, for adults, inpatient duration of therapy was shorter than outpatient duration.

While all observed safety end point outcomes occurred higher in the inpatient setting, there were no statistically significant differences between groups. Mortality was evaluated in 8 studies (RR .54, 95% CI .23–1.26, *P* = .16, I^2^ 18%. 2 pediatric, 6 adult RCTs). Treatment failure was evaluated in 11 studies (RR 1.0, 95% CI .59–1.72, *P* = .99, I^2^ 38%. 4 pediatric, 7 adult RCTs). ADRs were evaluated in 6 studies (RR .89, 95% CI .69–1.15, P = .38, I^2^ 0%. 2 pediatric, 4 adult RCTs). Device complications were reported in all studies (RR .58, 95% CI .17–1.98, P = .39, I^2^ 0%). Additionally, differing OPAT models of care (self-OPAT, caregiver-OPAT, and healthcare provider-OPAT) did not impact results.

This meta-analysis reinforces the notion that OPAT is non-inferior to traditional inpatient administered IV treatment. However, this study also demonstrates the paucity of data from controlled studies comparing inpatient versus outpatient therapy, both in terms of patient numbers and infections treated.

#### Making the EHR work for you-modifications of an electronic health record system to improve tracking and management of patients receiving outpatient parenteral antibiotic therapy

This study was a descriptive overview of a multimodal intervention centered around the EHR (Epic), to address gaps in care while safely and effectively monitoring a large patient volume at one facility in the USA.^12^ The EHR modifications created shared patient lists, standardized order sets, SmartForm-enabled documentation, automated notifications, and a consolidated “OPAT Monitoring View” which collated labs, microbiology, imaging, infusion details, and ID notes into a single screen, reducing cognitive load and rounding time.

These modifications facilitated consistent therapeutic drug monitoring, VAD oversight, and timely follow-up, while also generating extractable data for workload analysis and program justification. The authors report that multidisciplinary rounds averaged less than three hours per week while overseeing 130–145 patients per week. From 2019 to 2022, 3,402 OPAT episodes were supported (∼850/year), representing a greater than 50% increase in volume compared to 2016 including patients on high-risk oral and/or IV antibiotics requiring therapeutic drug monitoring.

Several issues addressed by the intervention, such as tracking lab results, poorly timed follow-up appointments, outstanding imaging results and coordination of care are familiar and frustrating to OPAT teams. Detailed descriptions, including snapshots of the actual workflow, will be useful to other OPAT programs that use Epic, from smaller practices to multicenter sites in a health system.

#### Exploring patients’ perspectives: a mixed methods study on Outpatient Parenteral Antimicrobial Therapy (OPAT) experiences

In an explanatory-sequential mixed-methods study design, Peter et al surveyed 58 patients and conducted 12 in-depth patient interviews about the patients’ experience with OPAT.^13^ The primary outcome of the study was to determine how OPAT is experienced by patients in an urban pilot area of Germany.

Most of the 58 survey respondents and half of the interview participants were male, and the mean age of survey respondents and interview participants was 55 years and 60 years, respectively. Mean duration of OPAT for survey respondents and interview participants was 15 days and 27 days, respectively. The most common indications for OPAT were bone and joint infections. All survey respondents rated their opinion of OPAT as very good (72.4%) or good (27.6%); all respondents rated the organization of OPAT treatment as very good (70.7%) or good (29.3%). Almost all patients would choose OPAT again (98.3%) and recommend OPAT (96.55%). Negative experiences with OPAT from the survey were infrequent but included problems with material storage, feeling uncomfortable with the VAD, the VAD affecting how they were perceived in public, and deliberately hiding the catheter in public. The interviews also revealed a positive opinion of OPAT, including the ability to be at home, leading to higher quality of life. Most interview participants expressed anxiety at the beginning of therapy about making mistakes when administering antibiotics. The challenges with OPAT expressed in the interviews included discomfort sleeping on the side of the catheter, financial challenges, and suspected side effects.

This study aligns with prior research showing patient preference for OPAT compared with staying in the hospital^14–16^ but also gives additional details about the problems patients receiving OPAT can experience.

#### Quantifying the time to administer outpatient parenteral antimicrobial therapy: a missed opportunity to compensate for the value of infectious diseases

In a prospective observational study with retrospective analysis, Schranz et al^17^ quantified OPAT work outside clinic visits, spanning ∼ 30 months at a large academic medical center in the USA. Time for common OPAT activities was self-collected, electronically captured, and extracted. Primary outcome was average “OPAT time” in minutes per week (min/wk) per OPAT case, stratified by time-based CPT (Current Procedural Terminology) code after censoring outlier episodes>15 weeks duration or >600 min OPAT time. Secondary outcomes were OPAT duration and clinic visits during OPAT.

1,064 home-based OPAT episodes yielded a median documented OPAT time of 27 min/wk per case. Vancomycin-containing regimens required 40 min/wk compared with 23 min/wk for non-vancomycin regimens. Presuming OPAT time per week can compress into a single weekly billable visit, 73% of episodes qualified for Level III billing, correlating to a $63 (facility) or $89 (non-facility) Medicare payment per week. Median OPAT duration was 35 days, with 12, 48, and 40% patients receiving 0, 1, and ≥ 2 follow-up appointments per OPAT course, respectively.

Although widely acknowledged that fee-for-service reimbursement remains inadequate compensation for OPAT services, few studies have attempted to quantify the time of complex multidisciplinary coordination required for managing OPAT patients outside visits. Beyond a focus on specific undervalued program aspects,^18,19^ this study promotes a holistic assessment (and billing crosswalk) of uncompensated OPAT labor, further accelerating the academic, administrative, and legislative conversations^20–25^ surrounding development of alternative reimbursement structures for quality- and value-laden programs such as OPAT.

### Therapy-based studies

#### Outcomes of ceftriaxone compared with cefazolin or nafcillin/oxacillin for outpatient therapy for methicillin-sensitive *Staphylococcus aureus* bloodstream infections: results from a large United States claims database

Ceftriaxone administered once daily is a practical regimen in patients receiving OPAT; however, its utility treating methicillin-susceptible *Staphylococcus aureus* (MSSA) bloodstream infections (BSI) is limited by little substantiating data. In this retrospective study,^26^ authors searched a 2010–2018 claims database to identify adults under 65 years old that received ceftriaxone versus cefazolin, nafcillin, or oxacillin (referred to herein as standard of care [SOC]) as OPAT for MSSA BSI. Patients discharged to skilled nursing facilities were excluded. Outcomes were infection-related and all-cause 90-day readmission rate.

Twenty-five percent (469/1895) of patients received ceftriaxone. More patients with pneumonia were prescribed ceftriaxone over SOC (22% vs 17.7%, *P* = .045). SOC was prescribed more than ceftriaxone in patients with epidural abscesses (11.9% vs 6.5%; *P* = .002) or endocarditis (15.9% vs 10.2%; *P* = .002). Patients prescribed SOC more frequently received an ID consultation or echocardiogram. The median OPAT duration was 15 days. Infection-related (17% vs 20%; adjusted odds ratio [aOR] .89 [95% CI, .67–1.18]) and all-cause 90-day readmission rates (25% vs 29%; aOR .79 [95% CI, .62–1.03]) were similar between ceftriaxone and SOC groups. The authors conclude ceftriaxone is a reasonable OPAT option for MSSA BSI. These results should be interpreted cautiously for high-risk or complicated cases and verified through a randomized clinical trial.

#### Dalba got back? Use of dalbavancin for the treatment of vertebral osteomyelitis

Off-label dalbavancin usage for osteomyelitis treatment has been reported in the literature.^27^ Streifel et al, describe utilization and treatment outcomes using dalbavancin specifically for vertebral osteomyelitis (VO), in a retrospective cohort at one facility in the USA.^28^

34 patients had VO diagnosed by a combination of radiographic, clinical, and intraoperative findings. 11 were prescribed a single 1,500 mg dalbavancin dose; 23 were prescribed two doses given one week apart, to achieve a 6-week duration. 17 of the single or first doses were administered in the inpatient setting; the remainder were administered in an infusion center or through a home infusion vendor. 27 (79.4%) patients had substance use history, 23 with injection drug use. *Staphylococcus aureus* (SA) was isolated in 22 (65%); 12 were methicillin-resistant SA. 11 patients had bacteremia, and 22 had epidural involvement. Source control procedure occurred in 15 patients. Hardware was present in 8, including 5 with new hardware placed. The most common reasons for dalbavancin usage were IVDU (24, 70.6%), lack of safe discharge environment (6), and prior medication non-adherence (6). Overall, 10 of 11 (90.1%) of the single dose patients completed therapy; 19 of 23 (82.6%) completed 2 doses. 30-day infection relapse/recurrence occurred in 2 patients; 90-day relapse/recurrence occurred in 1 patient. Overall, 29 of 34 patients (85.3%) completed the planned course.

Authors describe effective dalbavancin usage for VO treatment, in a cohort where 79% of the patients had a history of substance use disorders. Dalbavancin offers an encouraging alternative for VO treatment.

## Discussion

A multidisciplinary group of OPAT clinicians summarized ten OPAT publications from 2024, as voted on by a national multidisciplinary group of OPAT clinicians. Common themes that emerged from the top 10 articles included patient perspectives of OPAT, OPAT clinician workload, tools for OPAT work, use of ceftriaxone for MSSA BSI, and dalbavancin use. Compared with the 2023 top 10, factors for dalbavancin use remained a common theme. Based on the top 10 articles of 2024, remaining literature gaps determining the ideal laboratory monitoring frequency based on antimicrobial regimen. Limitations of this study include relying on the GOAT score, which incorporates article citations. Therefore, articles that had not yet been cited are not included in the Top 10 list. Another future area of study would be to explore different methods of determining a Top 10 list of published articles.
